# All Central Nervous System Neuro- and Vascular-Communication Channels Are Surrounded With Cerebrospinal Fluid

**DOI:** 10.3389/fneur.2021.614636

**Published:** 2021-06-17

**Authors:** Lara M. Fahmy, Yongsheng Chen, Stephanie Xuan, E. Mark Haacke, Jiani Hu, Quan Jiang

**Affiliations:** ^1^Department of Psychiatry and Behavioral Neurosciences, Wayne State University, Detroit, MI, United States; ^2^Department of Neurology, Wayne State University, Detroit, MI, United States; ^3^Department of Radiology, Wayne State University, Detroit, MI, United States; ^4^Department of Neurology, Henry Ford Health System, Detroit, MI, United States

**Keywords:** cerebrospinal fluid, central nervous system, glymphatic clearance, cerebral waste clearance, magnetic resonanace imaging

## Abstract

**Background:** Recent emerging evidence has highlighted the potential critical role of cerebrospinal fluid (CSF) in cerebral waste clearance and immunomodulation. It is already very well-established that the central nervous system (CNS) is completely submerged in CSF on a macro-level; but to what extent is this true on a micro-level? Specifically, within the peri-neural and peri-vascular spaces within the CNS parenchyma. Therefore, the objective of this study was to use magnetic resonance imaging (MRI) to simultaneously map the presence of CSF within all peri-neural (cranial and spinal nerves) and peri-vascular spaces *in vivo* in humans. Four MRI protocols each with five participants were used to image the CSF in the brain and spinal cord. Our findings indicated that all CNS neuro- and vascular-communication channels are surrounded with CSF. In other words, all peri-neural spaces surrounding the cranial and spinal nerves as well as all peri-vascular spaces surrounding MRI-visible vasculature were filled with CSF. These findings suggest that anatomically, substance exchange between the brain parenchyma and outside tissues including lymphatic ones can only occur through CSF pathways and/or vascular pathways, warranting further investigation into its implications in cerebral waste clearance and immunity.

## Introduction

The critical involvement of cerebrospinal fluid (CSF) in metabolic cerebral waste clearance (CWC) has gained a lot of momentum lately. Our traditional understanding of CSF circulation is that it is produced by the choroid plexus and circulates through the ventricles and subarachnoid spaces, exiting directly into the dural venous sinuses via arachnoid granulations/villi (1–5). Recent studies have expanded upon this traditional understanding and demonstrated that CSF penetrates and interacts with the brain parenchyma through a defined pathway with the purpose of CWC; this has been coined the glymphatic pathway (6). A significant portion of the subarachnoid CSF penetrates the brain parenchyma through para-arterial routes, facilitated by aquaporin-4 protein water channels (AQP4) on astrocytic end-feet (7–12). CSF then mixes with the interstitial fluid, and together, they are cleared debatably though para-venous routes with any associated solutes; once again, facilitated by AQP4 (8, 13–15). Dysfunctions of this glymphatic pathway have been associated with a broad range of neurological diseases including, but not limited to, Alzheimer's disease, stroke, traumatic brain injury, multiple sclerosis, diabetes, and chronic traumatic encephalopathy; begging the question of its involvement in virtually all neurodegenerative diseases (8–10, 12, 14, 16–19). Therefore, it has become apparent that gaining a complete and accurate understanding of CSF physiology is an essential prerequisite to understanding the pathophysiology underlying this broad range of neurological disease.

In light of the importance of CSF physiology, researchers have focused their attention on investigating CWC CSF outflow pathways, out of the central nervous system (CNS). The traditional outflow pathway via the arachnoid granulations/villi is becoming increasingly unpopular due to the lack of physiological evidence to support it (20, 21). This traditional pathway was primarily based on a study published over a 100 years ago, performed under non-physiologically high CSF pressure in human cadaver brain (22). More recently, the lymphatics have been identified as a major outflow pathway via peri-neural spaces (23, 24). Several studies have demonstrated the presence of CSF tracer within the sheaths surrounding cranial and spinal nerves; with the spotlight focused on the olfactory nerve (CN I) peri-neural space depositing into the nasal submucosa lymphatics (23–29). CSF within these peri-neural spaces exits the cranial cavity and vertebral column along the cranial and spinal nerves, respectively. At this point, CSF within the peri-neural spaces is proposed to be absorbed into the surrounding interstitial space/epidural tissue, and/or directly into the surrounding regional lymphatic vessels outside the CNS (26, 28, 30–34). Furthermore, the recently established meningeal lymphatic vessels are proposed to serve as another lymphatic-associated outflow pathway; However, the exact mechanism remains unclear (11, 35–37).

Although our understanding of CSF physiology remains relatively rudimentary and is still very much under investigation, one concept has become increasingly apparent: all CNS neuro- and vascular-communication channels are surrounded with CSF. It is already very well-established that the CNS is completely submerged in CSF on a macro-level; but now we know that this is true even on a micro-level, specifically within the peri-neural and peri-vascular spaces within the CNS parenchyma. Most studies have asynchronously investigated these spaces and these investigations have been mainly conducted in animals and human cadavers. Moreover, although CSF-filled peri-vascular spaces in several brain regions have been illustrated in several studies, to our knowledge, there is no systematic study focusing on whether all cerebral vasculature (with the exception of capillaries) are surrounded with CSF, and this can only be revealed using *in vivo* imaging methods. Therefore, the objective of this study was to use magnetic resonance imaging (MRI) to simultaneously map the presence of CSF within all peri-neural (cranial and spinal nerves) and peri-vascular spaces *in vivo* in humans.

## Method

To prove the concept, we designed four types of experiments each with five healthy participants (ages 23–59 years; three males and two females). Each protocol was tailored to a specific area of investigation and anatomical structures, such as cranial nerves and their corresponding perineural spaces, cerebral vasculature and their corresponding perivascular spaces, and different segments of the spinal cord. The study was approved by local Institutional Review Board. Written consent was obtained prior to each MRI experiment. Various sequences were used to image CSF in the brain and spinal cord at 3T (Verio, Siemens Healthineers, Erlangen, Germany).

**Figure 1:**
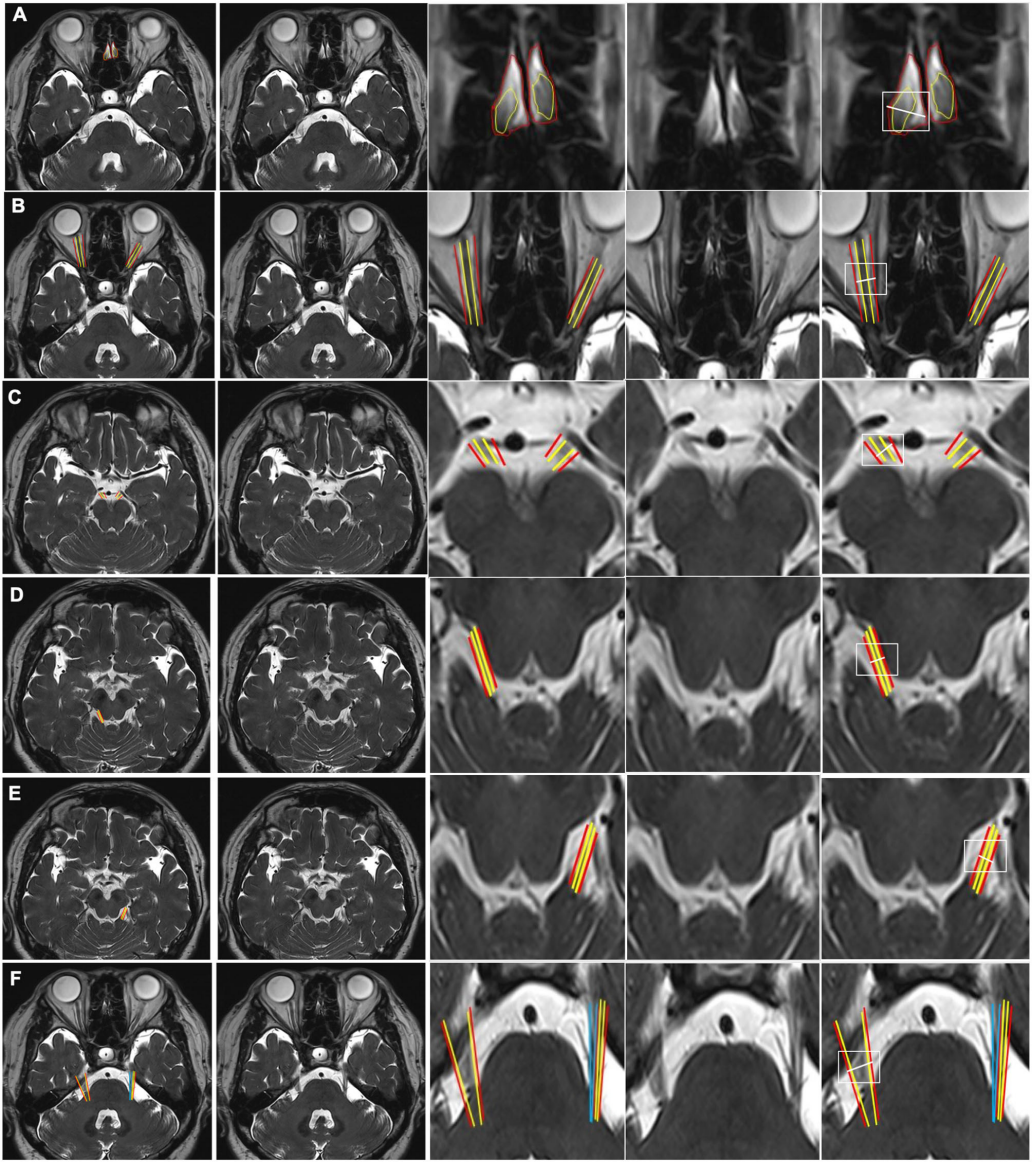
CSF-filled peri-neural spaces surrounding the first five cranial nerves (CN I-CN V). **(A)** Olfactory nerves (CN I), (two original images, two corresponding magnified images and measurement location); **(B)** Optic nerves (CN II) (two original images, two corresponding magnified images and measurement location); **(C)** Oculomotor nerves (CN III) (two original images, two corresponding magnified images and measurement location); **(D)** Right trochlear nerve (CN IV) (two original images, two corresponding magnified images and measurement location); **(E)** Left CN IV (two original images, two corresponding magnified images and measurement location); and **(F)** Trigeminal nerves (CN V) (two original images, two corresponding magnified images and measurement location). Space within the yellow lines: respective nerve (hypointense signal). Space between the red and yellow lines: peri-neural space (hyperintense signal). Space between the blue lines: CN V (hypointense signal). White line: measurement location. All images were generated from the first experiment as described in the method section.

**Figure 2:**
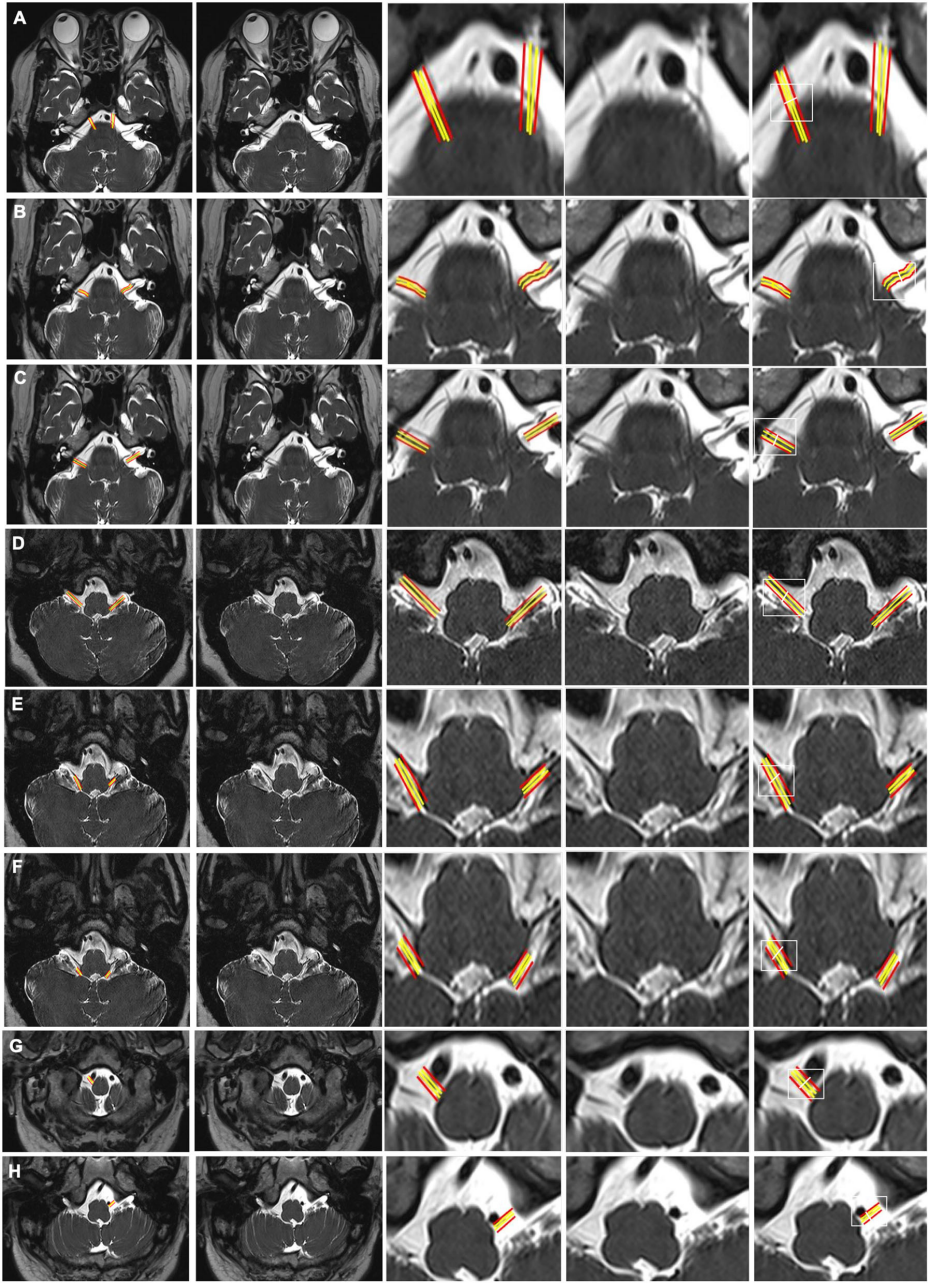
CSF-filled peri-neural spaces surrounding the cranial nerves 6–12 (CN VI-CN XII). **(A)** Abducens nerves (CN VI) (two original images, two corresponding magnified images and measurement location); **(B)** Facial nerves (CN VII) (two original images, two corresponding magnified images and measurement location); **(C)** Vestibulocochlear nerves (CN VIII) (two original images, two corresponding magnified images and measurement location); **(D)** Glossopharyngeal nerves (CN IX) (two original images, two corresponding magnified images and measurement location); **(E)** Vagus nerves (CN X) (two original images, two corresponding magnified images and measurement location); **(F)** Accessory nerves (CN IX) (two original images, two corresponding magnified images and measurement location); **(G)** Right hypoglossal nerve (CN XII) (two original images, two corresponding magnified images and measurement location); and **(H)** Left CN XII (two original images, two corresponding magnified images and measurement location). Space between the red and yellow lines: peri-neural space (hyperintense signal). White line: location to measure the diameters of cranial nerves and corresponding peri-neural spaces. All images were generated from the first experiment as described in the method section.

The first experiment employed a 3D T2-weighted (T2W) “**S**ampling **P**erfection with **A**pplication optimized **C**ontrasts using different flip angle **E**volution” (T2W-SPACE) sequence to image the CSF and cranial nerves with hyperintense CSF signal and hypointense signal for cranial nerves and blood vessels (38). This sequence yields a high nerve-to-CSF contrast and a high spatial resolution within a practical scanning time period. By optimizing timing parameters, uniform signal intensity of gray matter and white matter was acquired with repetition time (TR)/echo time (TE) = 1,000/132 ms. Imaging resolution was 0.5 × 0.5 × 0.8 mm^3^. With a parallel imaging accelerating factor of 2, the scanning time was 5 min for 114 axial slices covering the brain. All diameters were measured on the best shown slice using the standard software provided with the MRI scanner. We have reported the diameters as mean(±SE), as well as the diameter ratio of the cranial nerve diameter over the total diameter (cranial nerve diameter + corresponding peri-neural space diameter), for all 12 cranial nerves in healthy humans (Figures 1, 2).

For the second experiment, in order to image CSF in peri-vascular spaces and to demonstrate that all MRI-visible cerebral blood vessels were surrounded by CSF, we used high-resolution “**ST**rategically **A**cquired **G**radient **E**cho” (STAGE) imaging to simultaneously obtain bright-blood and dark-blood images (39–41). The STAGE imaging included a proton density weighted (PDW) scan and a T1-weighted (T1W) scan. Both images were fully flow compensated with bright blood signal. By subtracting the PDW from the inverted T1W image, one could obtain a synthetic T2W dark-blood image (sT2W) presenting bright-CSF and uniform gray matter and white matter intensities. The high-resolution STAGE data were acquired in the sagittal plane with a scan matrix of 2,048 × 832 leading to a resolution of 0.1 × 0.2 × 2.0 mm^3^. Scan time was 17 min. The STAGE method simultaneously yielded naturally co-registered dark-blood (sT2W) and bright-blood (T1W) which were used to visualize the blood vessels and perivascular spaces in the order of 100 μm. In addition, to acquire a pure CSF image with suppressed blood signals, we used a 3D turbo-spin echo (TSE) sequence with TR/TE = 2,000/345 ms and an isotropic voxel size of 0.5 mm. With such a long echo time, the images were heavily T2-weighted such that all tissues other than CSF (very long T2) were suppressed. Given the long TR/TE and high-resolution 3D sampling scheme, the sequence took half an hour covering the central half of the brain in the sagittal plane.

The third experiment was performed on the cervical spinal cord at the C4/C5 level. A 2D T2^*^ weighted spoiled gradient echo sequence (**M**ultiple **E**cho **D**ata **I**mage **C**ombination, MEDIC) was used to acquire images with hyperintense CSF and blood vessels, and hypointense nerves. This sequence is one of the clinical sequences for cervical protocol. Imaging parameters were: TR = 587 ms, TE = 17 ms, flip angle = 30°, in-plane resolution = 0.5 × 0.5 mm^2^, slice thickness = 3 mm, scanning time = 3 min.

The fourth and final experiment was for imaging the lumbar spinal nerves at L3/L4 level. A regular T2W-TSE sequence was used with the following imaging parameters: TR = 5,490 ms, TE = 87 ms, in-plane resolution = 0.6 × 0.6 mm^2^, slice thickness = 4 mm, scanning time = 2 min. This sequence, with sub-millimeter in-plane resolution, provided high nerve-to-CSF contrast. The 12 pairs of cranial nerves and spinal nerves were labeled by experienced neuroradiologist on images from the first, third and fourth experiments.

## Results

**Figure 3:**
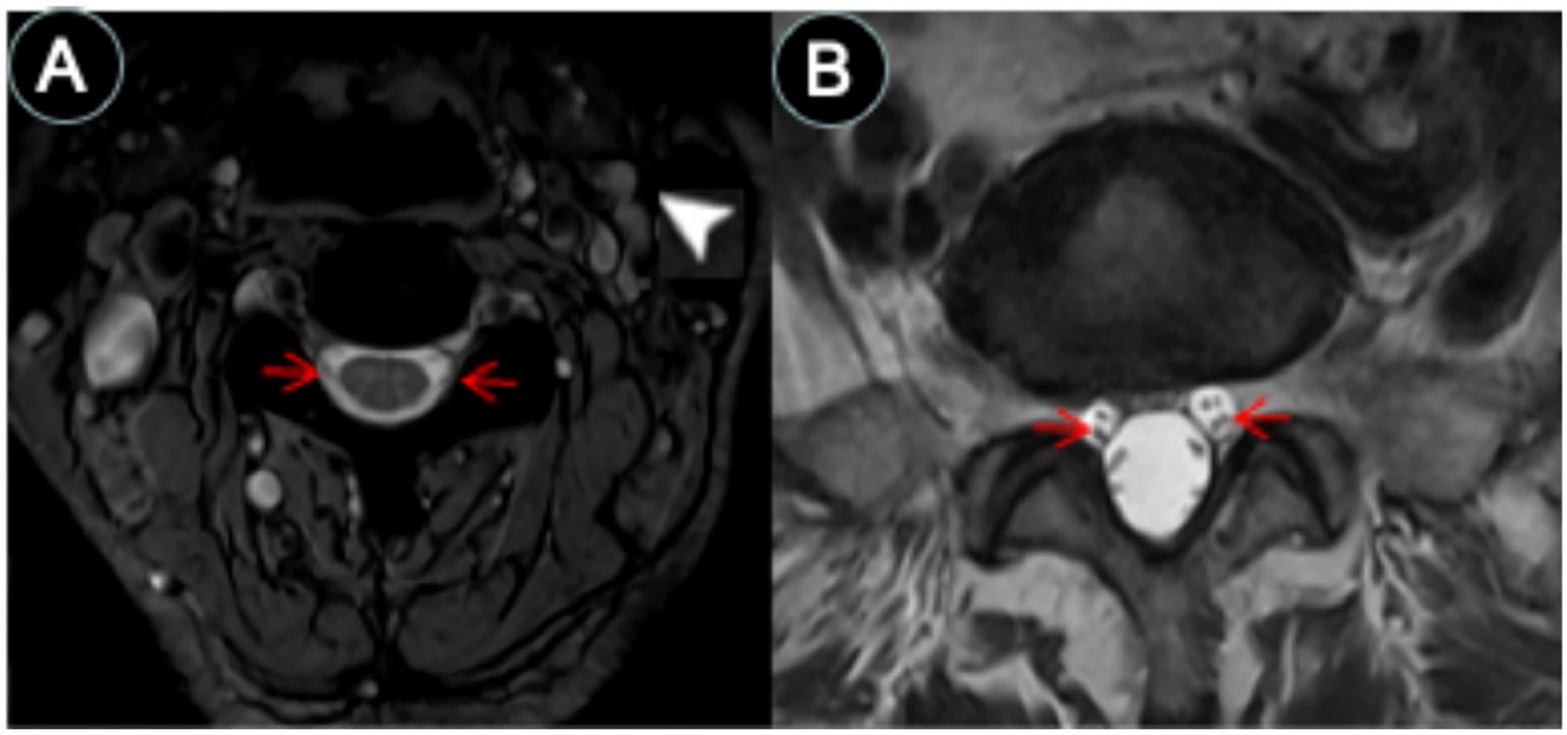
CSF-filled peri-neural spaces surrounding spinal nerves. **(A)** Cervical spinal nerves (brachial plexus). Cervical nerves four are pointed with red arrows (hypointense signal), and the space surrounding the nerves is the CSF-filled peri-neural space (hyperintense signal). Multiple lymph nodes are detected from MRI (white arrowhead). **(B)** Lumbar spinal nerves (red arrows, hypointense signal) surrounded with CSF-filled peri-neural space (hyperintense signal). Images were generated from the third and the fourth experiments as described in the method section.

**Figure 4:**
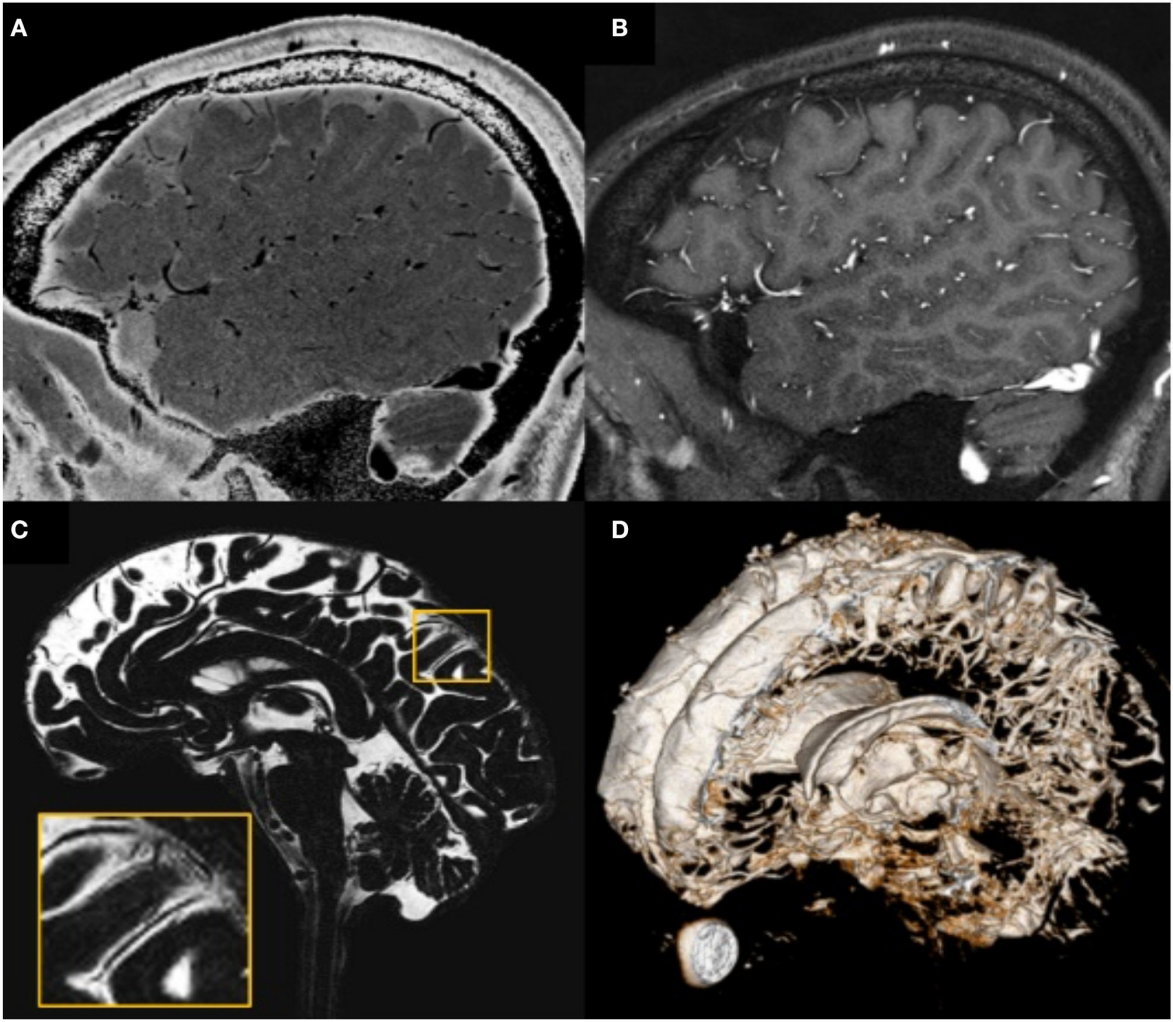
Illustration showing that all MRI-visible vasculature were surrounded by CSF. **(A)** sT2W image with bright CSF and dark blood; **(B)** T1W with bright blood signal but dark CSF signal; **(C)** CSF-only images, acquired by T2-TSE sequence with TE = 345 ms at 3T; **(D)** a 3D rendering of the CSF-only images. **(A)** and **(B)** were results from the high-resolution STAGE scans. The enlarged image in **(C)** shows several MRI-visible vasculature (dark curves) surrounded by bright CSF. Images were generated from the second experiment as described in the method section.

All MRI data were first visually evaluated to label nerves and vessels on representative images. Representative images of the first experiment are shown in Figures 1, 2. Images in Figure 3 are from the third and fourth experiments. The results of the second experiment are shown in Figure 4.

### The CNS Neuro-Communication Channels Are Surrounded With CSF

The CNS neuro-communication channels consist of 12 pairs of cranial nerves and 31 pairs of spinal nerves. Images from the first experiment were used to visualize all 12 pairs of the cranial nerves (Figures 1, 2). As illustrated, the cranial nerves were hypointense, while the peri-neural spaces surrounding the cranial nerves were hyperintense, indicating the presence of CSF within these spaces. We qualitatively observed that the peri-neural spaces had a slightly higher signal intensity than other CSF compartments. This is consistent with an array of previous studies which has already taken a closer look at the peri-neural spaces and its fluid contents, indicating that the peri-neural spaces are filled with a mixture of CSF and interstitial fluid (ISF) (24, 26–29, 42–51).

Figure 1A shows an axial view of a pair of hypointense CN I, encased in hyperintense CSF-filled perineural space, on either side of a boney projection inferior to the frontal lobe, the crista galli. The mean diameter for CN I was 4.3(±0.8) mm and the mean total diameter (CN I + corresponding peri-neural space) was 6.0(±0.6) mm, with a diameter ratio (CN I diameter over total diameter) of 0.7 (Table 1). Figure 1B shows an axial view of a pair of hypointense optic nerves (CN II), encased in hyperintense CSF-filled perineural space, emerging from the optic chiasm. The mean diameter for CN II was 3.2(±0.5) mm and the mean total diameter (CN II + corresponding peri-neural space) was 5.0(±0.5) mm, with a diameter ratio (CN II diameter over total diameter) of 0.6 (Table 1). Figure 1C shows an axial view of a pair of hypointense oculomotor nerves (CN III), surrounded with hyperintense CSF-filled perineural space, emerging from the midbrain. The mean diameter for CN III was 1.9(±0.5) mm and the mean total diameter (CN III + corresponding peri-neural space) was 2.6(±0.4) mm, with a diameter ratio (CN III diameter over total diameter) of 0.7 (Table 1). Figures 1D,E show axial views of a pair of hypointense trochlear nerves (CN IV), surrounded with hyperintense CSF-filled perineural space, emerging from the midbrain. The mean diameter for CN IV was 0.9(±0.3) mm and the mean total diameter (CN IV + corresponding peri-neural space) was 1.4(±0.5) mm, with a diameter ratio (CN IV diameter over total diameter) of 0.6 (Table 1). Figure 1F shows an axial view of a pair of hypointense trigeminal nerves (CN V), encased in hyperintense CSF-filled perineural space, emerging from the pons. The mean diameter for CN V was 4.6(±0.4) mm and the mean total diameter (CN V + corresponding peri-neural space) was 5.7(±0.4) mm, with a diameter ratio (CN V diameter over total diameter) of 0.8 (Table 1).

**Table 1:** Diameter measurements and diameter ratios for all 12 cranial nerves.

**Cranial nerve**	**Nerve diameter, mm mean(±SE)**	**Total diameter (nerve + corresponding peri-neural space), mmmean(±SE)**	**Ratio (nerve diameter over total diameter)**
CN 1	4.3(±0.8)	6.0(±0.6)	0.7
CN II	3.2(±0.5)	5.0(±0.5)	0.6
CN III	1.9(±0.5)	2.6(±0.4)	0.7
CN IV	0.9(±0.3)	1.4(±0.5)	0.6
CN V	4.6(±0.4)	5.7(±0.4)	0.8
CN VI	1.1(±0.1)	1.9(±0.2)	0.6
CN VII	1.0(±0.2)	1.6(±0.1)	0.6
CN VIII	1.1(±0.3)	1.7(±0.2)	0.6
CN IX	1.2(±0.1)	1.9(±0.3)	0.6
CN X	1.4(±0.2)	1.9(±0.3)	0.7
CN XI	1.1(±0.1)	1.7(±0.2)	0.6
CN XII	0.9(±0.2)	1.3(±0.1)	0.7

*Measurement locations are indicated in Figures 1, 2. We have reported the diameters as mean(±SE), as well as a diameter ratio of the nerve diameter over the total diameter (nerve diameter + corresponding peri-neural space diameter), for all 12 cranial nerves in five healthy human volunteers*.

Figure 2A shows an axial view of a pair of hypointense abducens nerves (CN VI), surrounded with hyperintense CSF-filled perineural space, emerging from the pons. The mean diameter for CN VI was 1.1(±0.1) mm and the mean total diameter (CN VI + corresponding peri-neural space) was 1.9(±0.2) mm, with a diameter ratio (CN VI diameter over total diameter) of 0.6 (Table 1). Figure 2B shows an axial view of a pair of hypointense facial nerves (CN VII), surrounded with hyperintense CSF-filled perineural space, emerging from the pons. The mean diameter for CN VII was 1.0(±0.2) mm and the mean total diameter (CN VII + corresponding peri-neural space) was 1.6(±0.1) mm, with a diameter ratio (CN VII diameter over total diameter) of 0.6 (Table 1). Figure 2C shows an axial view of a pair of hypointense vestibulocochlear nerves (CN VIII), surrounded with hyperintense CSF-filled perineural space, emerging from the pons. The mean diameter for CN VIII was 1.1(±0.3) mm and the mean total diameter (CN VIII + corresponding peri-neural space) was 1.7(±0.2) mm, with a diameter ratio (CN VIII diameter over total diameter) of 0.6 (Table 1). Figure 2D shows an axial view of a pair of hypointense glossopharyngeal nerves (CN IX), surrounded with hyperintense CSF-filled perineural space, emerging from the medulla. The mean diameter for CN IX was 1.2(±0.1) mm and the mean total diameter (CN IX + corresponding peri-neural space) was 1.9(±0.3) mm, with a diameter ratio (CN IX diameter over total diameter) of 0.6 (Table 1). Figure 2E shows an axial view of a pair of hypointense vagus nerves (CN X), encased in hyperintense CSF-filled perineural space, emerging from the medulla. The mean diameter for CN X was 1.4(±0.2) mm and the mean total diameter (CN X + corresponding peri-neural space) was 1.9(±0.3) mm, with a diameter ratio (CN X diameter over total diameter) of 0.7 (Table 1). Figure 2F shows an axial view of a pair of hypointense accessory nerves (CN IX), encased in hyperintense CSF-filled perineural space, emerging from the medulla. The mean diameter for CN XI was 1.1(±0.1) mm and the mean total diameter (CN XI + corresponding peri-neural space) was 1.7(±0.2) mm, with a diameter ratio (CN XI diameter over total diameter) of 0.6 (Table 1). Figures 2G,H show axial views of a pair of hypointense hypoglossal nerves (CN XII), encased in hyperintense CSF-filled perineural space, emerging from the medulla. The mean diameter for CN XII was 0.9(±0.2) mm and the mean total diameter (CN XII + corresponding peri-neural space) was 1.3(±0.1) mm, with a diameter ratio (CN XII diameter over total diameter) of 0.7 (Table 1).

The spinal nerves were visualized on the images from the third and fourth experiments (Figure 3). Similarly, the spinal nerves were hypointense, while the peri-neural spaces surrounding the spinal nerves were hyperintense, indicating the presence of CSF. Figure 3A shows an axial view of a pair of hypointense cervical spinal nerves emerging from the spinal cord at the level of C4/C5, surrounded with hyperintense CSF-filled perineural space. Figure 3B shows an axial view of a pair of hypointense lumbar spinal nerves emerging from the spinal cord at the level of L3/L4, encased in hyperintense CSF-filled perineural space.

### The CNS Vascular-Communication Channels Are Surrounded With CSF

The high-resolution (in the order of 100 μm) sT2W (Figure 4A, bright CSF and dark blood) and T1W (Figure 4B, bright blood and dark CSF) derived from STAGE imaging demonstrated that all MRI-visible vasculature were surrounded with CSF. By comparing these two images, we found that all MRI-visible vasculature were indeed surrounded by CSF. Moreover, in order to further confirm these findings, we used a T2W-TSE image acquired with a very long echo time (TE = 345 ms at 3T) to generate “pure” CSF images, with bright CSF and dark blood (Figures 4C,D). Once again, all MRI-visible vasculature were indeed surrounded with CSF.

## Discussion

To our knowledge, this is the first study to simultaneously and systematically verify that all 12 pairs of cranial nerves, all MRI-visible vasculature and spinal nerves are surrounded with CSF *in vivo* in humans; as opposed to previous studies that have asynchronously investigated this, mainly in animals and human cadavers. Our findings indicate that all brain parenchyma and spinal cord communication channels, both neuro- and vascular-communication channels, are encased in CSF. The diameter ratios, nerve diameter over total diameter (nerve diameter + corresponding peri-neural space), ranged from 0.6–0.8. It logically follows that CSF must play a critical role in substance exchange between the brain parenchyma/spinal cord and its communication channels as well as its surrounding environment/tissue—a role that has been historically underestimated and understudied. Our findings are consistent with previous literature that have asynchronously investigated CSF-filled peri-neural spaces, mostly in the context of CWC lymphatic-associated CSF outflow pathways, as well as peri-vascular spaces (2, 7, 8, 52, 53).

Our findings indicated that CN I is surrounded with a CSF-filled peri-neural space, with a diameter ratio of 0.7. This is consistent with several studies that have focused on investigating the peri-neural spaces surrounding CN I. In 1958, Svane-Knudsen et al. observed that an iron solution injected in the CSF of guinea pigs made its way to the peri-neural spaces of CN I and the nasal interstitium (43). Bradbury et al. found that a radio-labeled intra-parenchymal tracer injection in rabbits drained via peri-vascular spaces and peri-neural spaces surrounding the CN I, allowing passage into the nasal submucosa and lymphatics (45). Similar findings were observed in a study conducted by Pile-Spellman et al. in cats and rabbits, where radiolabeled colloid tracer injected in the ventricles made its way into the nasal submucosa and eventually into the cervical lymph nodes (46). A series of experiments conducted by Mollanji et al. identified the peri-neural spaces surrounding CNI as a major lymphatic-associated CSF outflow pathway in sheep; and demonstrated that blockage of the cribriform plate increased intracranial pressure and CSF outflow resistance, and decreased CSF transport (48–50). Moreover, in a series of experiments conducted by Johnston et al. in sheep, pigs, rabbits, mice, rats, monkeys and human cadavers, demonstrated that a silicone compound CSF tracer made its way into an extensive network of lymphatic vessels present in the nasal submucosa via peri-neural spaces surrounding CN I (28). More recent studies have been consistently confirming these findings (24, 29, 51). Furthermore, and interestingly, Czerniawska et al. found evidence of retrograde transport of a gold tracer injected in rabbit nasal mucosa back into the CSF (44). However, to the best of our knowledge, none of these studies have reported a quantitative diameter ratio for CN I.

Our findings also indicated that, in addition to CN I, the other 11 cranial nerves are also surrounded with CSF-filled peri-neural spaces, with diameter ratios ranging from 0.6–0.8. The peri-neural spaces of other cranial nerves have been studied to a lesser extent. In 1972, Arnold et al., using mice, rats, guinea pigs and rabbits, reported that the peri-neural spaces along CNV III played a role in CSF drainage into the perilymphatic spaces of the inner ear, following ink and thorotrast CSF cisterna magna tracer injection. Moreover, Kida et al. observed CSF tracer deposition along the peri-neural spaces of CN II, in addition to CN I and CN III, following an injection of india ink CSF cisterna magna tracer in rats (26). Similar findings (for CN I, CN II and CNV III) were observed in an experiment conducted by Boulton et al. in sheep injected with radiolabeled CSF tracer (47). Furthermore, Zakharov et al. identified the peri-neural spaces along CN V, CNV II, CN IX, CN X, CN XII and spinal nerves as contributors to the lymphatic-associated CSF outflow pathway in sheep, following a silicone compound CSF cisterna magna injection (27). A couple of other studies have also identified the spinal nerves as contributors to the lymphatic-associated CSF outflow pathway (29, 42). More recently, Ma et al. identified the peri-neural spaces along CN XI, in addition to CN I, CN II, CN V, CN VII, CN IX and CN X, as contributors to the lymphatic-associated CSF outflow pathway in rats, following a near-infrared CSF lateral ventricle injection (24). To our knowledge, the peri-neural spaces along the CN III, the CN IV and CN VI have not been previously identified as CSF-filled and potentially contributing to lymphatic-associated CSF outflow pathways. Moreover, to our knowledge, none of these previous studies have reported quantitative diameter ratios for the cranial nerves. Unlike the diameter ratios of peri-vascular spaces, which have been previously reported mainly in animal studies (8), the diameter ratios of peri-neural spaces have simply passed under the radar. The utility of these diameter ratios can be investigated in neurodegenerative diseases such as Alzheimer's disease. In other words, future studies can focus on investigating the association between these diameter ratio changes and neurodegenerative diseases; since enlarged peri-vascular spaces have been reported with neurodegenerative diseases (17, 54, 55).

Our findings drive home the point that CSF lays between every cranial/spinal nerve and its surroundings (i.e., brain parenchyma and/or peripheral lymphatics), in the peri-neural spaces. Therefore, CSF must play a critical role in regulating the communication between the cranial nerves and their surroundings. One such type of communication regulation is with the lymphatic system. In fact, the interaction of CSF with the lymphatic system is only beginning to be understood. There is a growing body of evidence to suggest that CSF tracer eventually makes it into the deep cervical lymph nodes (24, 36, 37). Historically, the CNS is considered an immune-privileged site and therefore it was believed that antigens in the CNS did not communicate with the immune system (56–58). However, about 14–47% of radiolabeled tracers make their way into the lymphatic system, following CSF or intra-parenchymal injections (59). Therefore, antigens are transported to the immune system and can induce an immune response (56–58). Louveau et al. further demonstrated a decrease in the inflammatory response of brain-reactive T cells in a multiple sclerosis mouse model, following meningeal lymphatic vessel ablation (37). Anatomically-speaking, there is no direct interaction between the lymphatic system and CNS, and any interaction can only be accomplished through the CSF. Therefore, this begs the question of the role of CSF in immunomodulation, and together with our findings, the magnitude of this role.

Our findings also drive home the point that all MRI-visible vasculature is surrounded with CSF-filled peri-vascular spaces. Therefore, CSF must play a critical role in regulating the communication between the blood and the brain parenchyma, as well as CWC at large. In 1985, Rennels et al. introduced the idea of a fluid circulation system throughout the CNS via peri-vascular spaces (7). But it was not until 2012 that Iliff et al. solidified our understanding of this CSF circulation system via peri-vascular spaces within the brain parenchyma (labeled the glymphatic pathway); and demonstrated its critical role in amyloid beta (Aβ) clearance (8). Further investigations have indicated that the diameter ratios between the peri-arterial spaces and their corresponding arterial vessels are larger than the diameter ratios between the peri-venous spaces and their corresponding venous spaces (60). As mentioned earlier, dysfunctions of this glymphatic pathway have been associated with a broad range of neurological diseases including, but not limited to, Alzheimer's disease, stroke, traumatic brain injury, multiple sclerosis, diabetes, and chronic traumatic encephalopathy; begging the question of its involvement in virtually all neurodegenerative diseases (8–10, 12, 14, 16–19). Therefore, disruptions to CWC can either initiate or/and aggravate the pathophysiology of an array of neurological diseases.

Starting 6 years ago, gadolinium-based contrast agent (GCA) residual deposition in the brain parenchyma with chronic administration has been documented (61, 62). GCA is a widely used contrast agent in clinical MRI. It does not cross the BBB and is renally eliminated from the human body; and thus, considered safe for clinical use. Therefore, the residual deposition of IV-administered GCA in individuals with an intact BBB and normal renal function raised a lot of questions. How does the IV-administered GCA enter and deposit in the brain parenchyma? Currently, the mainstream theory is through the CSF via the glymphatic pathway. Based on several studies, it is hypothesized that the IV-administered GCA enters the CSF compartment through the choroid plexus, cranial nerve endings and/or peri-vascular spaces surrounding vessels; from here it re-circulates and enters the brain parenchyma via peri-arterial routes—a portion gets deposited in the brain parenchyma, while the remaining portion gets carried away via peri-venous routes, eventually draining into the lymphatics (63–66). CSF involvement was further confirmed by Öner et al. in a study investigating individuals with chronic intra-thecal administration of GCA who had never undergone IV-administration of GCA; similar GCA deposition patterns were observed (67). GCA deposition via CSF is an example of how CSF-filled peri-vascular and peri-neural spaces play a critical and major role in substance deposition and re-circulation. Long-term effects of GCA deposition are unknown and require further investigation.

There are several major strengths to our study. First, to our knowledge, it is the first study to systematically and simultaneously verify that CSF surrounds all cranial (including CN III, CN IV and CN VI which have not been previously investigated), all MRI-visible vasculature and spinal nerves. Second, our study was performed *in vivo* in humans. Third, our study utilized MRI which is minimally invasive and therefore caused minimal disruption to the biological system. In the past, most MRI studies of the peri-neural and peri-vascular spaces have been mainly conducted in animals (64, 68).

On the other hand, the major limitation of our study is that we did not investigate the underlying mechanisms of how CSF made it into or out of these peri-vascular and peri-neural spaces. Although this has been a topic-of-interest as of late, and several strides have been made to address this topic, the latest of which has been the establishment of the glymphatic pathway, the CSF efflux mechanisms remain unclear. One current question of interest is: “What is the underlying pathway/mechanism by which CSF drains into the meningeal lymphatics, and how is this pathway/mechanism related to CSF-filled peri-vascular and peri-neural spaces?” Another major limitation is that we did not investigate if and how CSF communicates with the CNS neuro- and vascular-communication channels themselves (i.e., the vasculature and nerves that it surrounds), nor a quantitative morphometric measurement of the arterial and venous blood vessels and their corresponding peri-vascular spaces. There is growing evidence to suggest that CSF in peri-vascular spaces might be involved in the direct transport of cerebral waste into the parenchymal vasculature, perhaps through transcellular mechanisms (29, 69, 70). However, given the pathophysiological variations, the systematic measurement of blood vessels and their corresponding peri-vascular space across multiple regions of the brain are complex and also warrant further investigation. Another major limitation is our small sample size of healthy human volunteers. Therefore, future investigations with larger sample sizes are warranted; perhaps even these investigations should be extended to beyond healthy individuals, i.e., patients with neurogenerative diseases such as Alzheimer's disease. Finally, the measured size of cranial nerves and their relative perineural spaces may suffer from partial volume effects which is a common issue with MRI.

In conclusion, our findings indicate that all CNS neuro- and vascular-communication channels are surrounded with CSF. In other words, all peri-neural spaces surrounding the cranial and spinal nerves as well as all peri-vascular spaces surrounding MRI-visible vasculature were filled with CSF. These findings highlight the extent to which CSF infiltrates the parenchyma and its importance, therefore, warranting further investigation into its potential implications.

## Data Availability Statement

The raw data supporting the conclusions of this article will be made available by the authors, without undue reservation.

## Ethics Statement

The studies involving human participants were reviewed and approved by Wayne State University Institutional Review Board. The patients/participants provided their written informed consent to participate in this study.

## Author Contributions

LF participated in conceptualization, data acquisition, data analysis, manuscript writing, and manuscript editing. YC participated in conceptualization, investigation, data acquisition, data analysis, manuscript writing, and manuscript editing. SX participated in data acquisition, data analysis, and manuscript editing. EH participated in methodology, data analysis, and manuscript editing. JH participated in conceptualization, methodology, data analysis, and manuscript editing. QJ participated in conceptualization, methodology, data analysis, and manuscript editing. All authors contributed to the article and approved the submitted version.

## Conflict of Interest

The authors declare that the research was conducted in the absence of any commercial or financial relationships that could be construed as a potential conflict of interest.
